# Analytical evaluation of the performances of point-of-care and benchtop procalcitonin assays in comparison with the B⋅R⋅A⋅H⋅M⋅S PCT sensitive KRYPTOR assay

**DOI:** 10.3389/fmed.2025.1487557

**Published:** 2025-06-17

**Authors:** Ruiqing He, Xiaobing Sun, Ling Su, Ting Zhu, Jiong Wu, Qi Hou

**Affiliations:** Department of Clinical Laboratory, Shanghai Jiahui International Hospital, Shanghai, China

**Keywords:** procalcitonin, algorithms, respiratory tract infections, sepsis, comparison

## Abstract

**Background:**

Procalcitonin (PCT) is increasingly utilized in clinical laboratories, leading to the proliferation of commercial PCT assays. However, not all of these assays are traceable to the B⋅R⋅A⋅H⋅M⋅S PCT standard, which is integral to established PCT clinical algorithms. This study evaluates the suitability of three non-B⋅R⋅A⋅H⋅M⋅S PCT assays for the application of these algorithms.

**Methods:**

The study assessed PCT assays from Wondfo (PCT-W), Getein (PCT-G), and Snibe (PCT-S), comparing them to the B⋅R⋅A⋅H⋅M⋅S PCT sensitive KRYPTOR assay (PCT-KR). Analytical performance, including linearity, imprecision, and recovery, was evaluated. Additionally, a method comparison study involving 350 routine serum samples was conducted to assess agreement, bias, and correlation with the KRYPTOR assay.

**Results:**

The KRYPTOR assay exhibited a maximum imprecision of 4.65%, while Wondfo, Getein, and Snibe showed higher imprecision at 8.38, 10.25, and 15.67%, respectively. Wondfo and Getein assays exceeded the maximum allowable deviation from linearity, and the Snibe assay failed the recovery assessment. Passing-Bablok regressions for low-range samples indicated significant bias for Wondfo (PCT-W = 0.663 PCT-KR + 0.076) and Getein (PCT-G = 0.838 PCT-KR−0.06). Agreement with the KRYPTOR assay was Kc = 0.83 and Kc = 0.87 for Wondfo and Getein, respectively, with substantial agreement in lower respiratory tract infections (LRTI) at Kc = 0.78 and Kc = 0.65. The Snibe assay showed better overall agreement (PCT-S = 1.002 PCT-KR−0.069), with Kc = 0.92 for sepsis and Kc = 0.76 for LRTI.

**Conclusion:**

Despite high overall agreement with the KRYPTOR assay, the evaluated assays (Wondfo, Getein, and Snibe) exhibit insufficient analytical performance at low PCT concentrations, which may limit their reliability in the diagnosis and management of sepsis and LRTI.

## Introduction

Procalcitonin (PCT) is the prohormone precursor of calcitonin, encoded by the CALC-1 gene, which is primarily transcribed in thyroid C-cells that produce and secrete calcitonin (1). Under normal physiological conditions, PCT is present in minimal concentrations in the serum (2). However, following pro-inflammatory triggers, especially those related to bacterial infections, PCT production can become significant in various cell types throughout the body. PCT levels can rise rapidly, within 3 to 6 h, after a systemic bacterial insult (1, 3, 4). This rapid response has led to the adoption of PCT as a biomarker for infection, making it a valuable diagnostic tool for suspected bacterial infections (2, 5). Additionally, PCT has been increasingly used to guide antibiotic treatment in patients with lower respiratory tract infections (LRTI) and sepsis (6).

The World Health Organization has recognized the importance of procalcitonin (PCT) by including it in its list of essential diagnostics (7), and many international guidelines highlight its utility for various clinical applications (8–11). In China, several expert consensus documents have been developed, providing detailed guidance on the use of PCT. As early as 2012, the Chinese Expert Consensus on Emergency Clinical Application of Procalcitonin recommended the use of specific PCT cut-offs—0.1, 0.25, and 0.5 μg/L—for diagnostic and therapeutic assessments. Additionally, algorithms based on proportional changes in PCT concentrations of 30% and 90% were introduced (12). Overall, Chinese consensus documents emphasize the use of PCT in diagnosing sepsis (12–14) and lower respiratory tract infections (LRTI) (12, 15–17), guiding antibiotic treatment (12–17), and in pediatric and neonatal care (18, 19).

National and international guidelines recommend using assays traceable to the B⋅R⋅A⋅H⋅M⋅S PCT standard, as the clinical cut-offs and algorithms for PCT testing were originally established using B⋅R⋅A⋅H⋅M⋅S PCT assays (9). In China, PCT testing is widely available, with numerous assays offered both in point-of-care formats and on large, automated immunoassay analyzers (20). Over the years, several immunoassay manufacturers have obtained a B⋅R⋅A⋅H⋅M⋅S PCT license, ensuring alignment with the B⋅R⋅A⋅H⋅M⋅S PCT gold standard. Companies such as Abbott, bioMérieux, DiaSorin, Fujirebio, Hybiome, LSI Medience, Quidel Ortho, Roche, Siemens, and ThermoFisher Scientific all offer B⋅R⋅A⋅H⋅M⋅S PCT assays on their immunoassay platforms and participate in the B⋅R⋅A⋅H⋅M⋅S PCT harmonization program (9). However, many other PCT assays do not clearly define their traceability to the B⋅R⋅A⋅H⋅M⋅S PCT standard, and there is a lack of information regarding the alignment of these non-B⋅R⋅A⋅H⋅M⋅S assays with the B⋅R⋅A⋅H⋅M⋅S PCT gold standard (9).

This study aims to shed light on the alignment of non-B⋅R⋅A⋅H⋅M⋅S PCT assays with a B⋅R⋅A⋅H⋅M⋅S PCT assay, specifically addressing whether the same diagnostic criteria and algorithms can be applied to non-B⋅R⋅A⋅H⋅M⋅S assays. Three such PCT assays, commercialized by Wondfo, Getein, and Snibe, were selected for evaluation, with particular focus on their performance when applying the low-range cut-offs (0.1 μg/L and 0.25 μg/L) established in common guidelines. Wondfo, Getein, and Snibe were selected based on their dominant market shares in China’s POCT sector (28, 22, and 18%, respectively; 2023 data) and their manufacturer-claimed advantages: rapid turnaround (Wondfo: 15 min), automated platform compatibility (Getein), and broad analytical range (Snibe: 0.04 μg/L–100 μg/L).

## Materials and methods

### Instruments and reagents

Three commonly used immunoassays were compared with the B⋅R⋅A⋅H⋅M⋅S PCT sensitive KRYPTOR assay, which is performed on the KRYPTOR Compact Plus instrument (B⋅R⋅A⋅H⋅M⋅S GmbH, Hennigsdorf, Germany, abbreviated as PCT-KR). The assays compared were the Finecare PCT assay, run on the FS-114 analyzer (Guangzhou Wondfo Biotech, Guangzhou, China, abbreviated as PCT-W); the Getein Biotech Procalcitonin assay kit, run on the Getein 1600 analyzer (Getein Biotech, Nanjing, China, abbreviated as PCT-G); and the Maglumi PCT Assay Kit, performed on the Maglumi 4000 Plus analyzer (Snibe, Shenzhen, China, abbreviated as PCT-S). Unless otherwise specified, reagents and protocols provided in the instructions for use of each respective diagnostic assay were followed. The core technologies and formats of these assays differ: PCT-W and PCT-G are based on lateral flow immunofluorescence, with manual and automated sample handling, respectively (21, 22). In contrast, PCT-S is a chemiluminescence assay that utilizes isoluminol and fluorescein chemistry in combination with magnetic beads. The KRYPTOR assay is unique in that it does not involve a solid-phase step, instead employing time-resolved amplification cryptate emission (TRACE) technology (23).

### Linearity

Two sample pools were prepared: one with low PCT concentrations using samples from healthy subjects, and another with high PCT concentrations using samples from patients with elevated PCT levels. To create the linearity test samples, aliquots from the high concentration pool were diluted using the low concentration pool. Five scalar dilutions of the high concentration pool were used to generate five of the six linearity test samples, covering concentrations up to the upper limit of the direct measurement range for each assay. Additionally, a low concentration pool sample was tested. Each sample was measured in triplicate, and the resulting data were visually inspected. Linearity deviations were evaluated according to CLSI EP6, 2nd Edition, with a 15% allowable deviation from linearity (ADL). Pearson’s linear correlation coefficients (r) were calculated to assess alignment with manufacturer specifications. The measuring ranges for the three evaluated assays were as follows: Snibe (0.04 μg/L–100.0 μg/L), Wondfo (0.1 μg/L–100.0 μg/L), and Getein (0.1 μg/L–50.0 μg/L), with analytical detection limits of Snibe (0.04 μg/L), Wondfo (0.1 μg/L), and Getein (0.1 μg/L) (21, 22, 24).

### Imprecision

Three serum pools were prepared at concentrations approximating key PCT cut-offs for LRTI and sepsis: 0.25, 0.5, and 2 μg/L. Each sample was tested in triplicate over five consecutive days. Repeatability, measured as intraday imprecision, and reproducibility, reflecting total laboratory imprecision, were then calculated.

### Recovery

Following the China National Accreditation Service for Conformity Assessment (CNAS) guidelines (CNAS-GL037), single patient routine serum PCT samples were mixed with standard solutions. The volume of the standard solution added never exceeded 10% of the total sample volume. For each assay, two sample solutions with different concentrations of PCT were prepared using a single patient sample. These sample solutions were measured in triplicate, and the mean deviations from the target recovery concentrations were calculated. The target recovery values were established by adding the initially measured serum sample concentrations to the PCT concentration contributed by the standard solutions. The standard solution concentrations provided by the respective manufacturers were used in these calculations. Recovery results were considered acceptable if they fell within ± 10% of the target recovery value. The Snibe PCT High Calibrator Solution (Snibe, Shenzhen, China) was used as the standard solution for samples tested with PCT-S, while the remaining samples were prepared using the B⋅R⋅A⋅H⋅M⋅S PCT Sensitive KRYPTOR CAL standard solution (B⋅R⋅A⋅H⋅M⋅S, Hennigsdorf, Germany).

### Method comparison

A total of 350 serum samples were collected from patients aged 18–85 years (mean: 45.3 years; male: 52%, female: 48%) with suspected bacterial infections. Testing was conducted in parallel on all instruments within 24 h of sample arrival at the laboratory. All serum samples were centrifuged and separated within 2 h after collection, stored in a −80°C freezer without undergoing freeze-thaw cycles. Numerical results from samples yielding PCT-KR values between the limit of quantification (LoQ) and 2 μg/L, as well as up to 100 μg/L, were included in the bias and correlation analyses. Bias values of the new assays were compared with those obtained from PCT-KR using normalized Bland-Altman plots and Passing-Bablok regression analysis. Correlation was assessed using Spearman’s rank correlation coefficient (rS). Cohen’s Kappa coefficients (K_*C*_) were calculated to establish agreement with PCT-KR. When classifying patient samples, concentration ranges established for the diagnosis of LRTI and systemic bacterial infections and sepsis (see Table 1) were used. The interpretation of Kappa (KC) values followed the criteria established by Landis and Koch: KC < 0 indicates no agreement; KC = 0 to 0.20 represents slight agreement; KC = 0.21 to 0.40 signifies fair agreement; KC = 0.41 to 0.60 denotes moderate agreement; KC = 0.61 to 0.80 reflects substantial agreement; and KC = 0.81 to 1.0 indicates perfect agreement (25).

**TABLE 1:** Class categorization according to reference PCT concentration ranges employed for LRTI and systemic bacterial infection-sepsis diagnosis (21–24).

LRTI classes	Systemic bacterial infection/ sepsis classes
Class 0: < 0.1 μg/L	Class 0: < 0.5 μg/L
Class 1: ≥ 0.1 μg/L, < 0.25 μg/L	Class 1: ≥ 0.5 μg/L, < 2 μg/L
Class 2: ≥ 0.25 μg/L, < 0.5 μg/L	Class 2: ≥ 2 μg/L, < 10 μg/L
Class 3: ≥ 0.5 μg/L	Class 3: ≥ 10 μg/L

## Results

### Linearity

**FIGURE 1:**
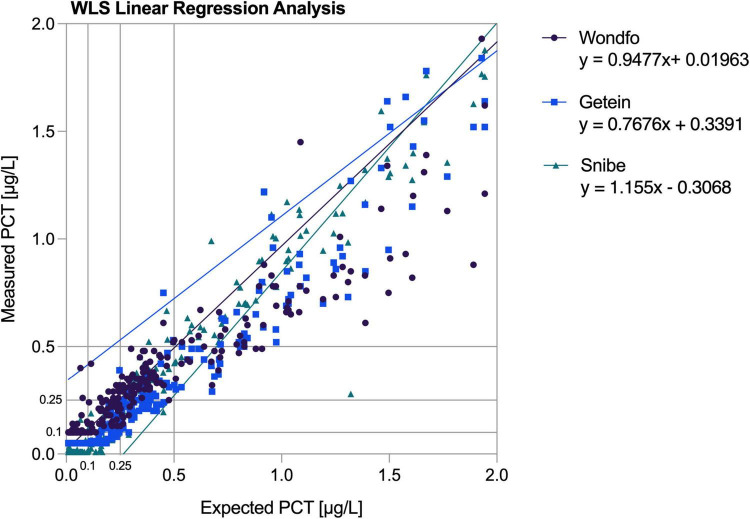
Linearity plots of the average measured values confronted with the predicted weighted least square regression line. WLS, weighted least square.

PCT-KR deviations did not exceed the ADL throughout the measured range while featuring a high linear correlation (*r* = 0.997). Deviations exceeding ADL were observed for both the PCT-W and PCT-G assays. PCT-W displayed deviations of −26% and −17% (respectively, corresponding expected values were 80.25 μg/L and 16.18 μg/L). A maximum-19% deviation (expected value: 31.54 μg/L) was observed in PCT-G’s assay (see Figure 1). PCT-W and PCT-G both displayed high linear correlations (respectively, *r* = 0.964 and *r* = 0.987). Despite producing a persistent readily visible deviation from the predicted regression line above 30 μg/L (see Figure 1), the PCT-S assay didn’t exceed the established ADL and its correlation coefficient was very high (*r* = 0.999).

### Imprecision

As displayed in Table 2, total laboratory imprecision for the PCT-KR assay is rather low and stable, ranging from 4.42% to 4.65% across concentrations, meanwhile, all other assays display higher imprecisions. The PCT-W and PCT-G assays showed total laboratory imprecision with coefficients of variation (CV) ranging from 6.07% to 8.38% and 8.57% to 10.25%, respectively. The PCT-S assay exhibited the highest total laboratory reproducibility CV, reaching 15.67%. Notably, the PCT-S assay demonstrated a significant increase in imprecision when comparing high and intermediate concentrations to the low concentration, with CVs of approximately 7.18% at ∼2 μg/L, 8.21% at ∼0.5 μg/L, and 15.67% at ∼0.25 μg/L.

**TABLE 2:** Imprecision results based on 15 measurements per sample and assay.

Assay	Sample	Mean	Repeatability CV	Reproducibility CV	95% CI % deviation
KRYPTOR	1 (∼0.25 μg/L)	0.248	2.08%	4.64%	3.1% to 12.5%
Wondfo		0.269	8.38%	8.38%	6.5% to 16.0%
Getein		0.297	8.57%	8.57%	6.8% to 15.4%
Snibe		0.249	10.71%	15.67%	11.4% to 38.5%
KRYPTOR	2 (∼0.5 μg/L)	0.418	2.39%	4.65%	3.2% to 12.3%
Wondfo		0.401	4.50%	6.07%	4.5% to 14.4%
Getein		0.493	6.20%	8.66%	6.4% to 20.9%
Snibe		0.413	3.05%	8.21%	5.3% to 22.6%
KRYPTOR	3 (∼2.0 μg/L)	2.065	2.77%	4.42%	3.1% to 11.2%
Wondfo		1.638	7.11%	8.02%	6.2% to 17.2%
Getein		1.977	8.73%	10.25%	7.8% to 22.6%
Snibe		1.955	2.09%	7.18%	4.5% to 20.1%

Repeatability and reproducibility refer, respectively, to intraday imprecision and total laboratory imprecision.

### Recovery results

The PCT-KR assay demonstrated good recovery performance, with values ranging from −0.79% to −3.83%. The PCT-S assay also showed favorable recovery for the low concentration sample (−1.87%). However, the other assays exhibited excessively high recovery values at various concentrations, exceeding the established ± 10% acceptance criterion (see Table 3).

**TABLE 3:** Summary of recovery assay results: original patient sample PCT concentrations according to the respective assays are featured in the mean control sample.

Assay	Mean recovery sample [μg/L]	Mean control sample [μg/L]	Standard sample [μg/L]	Volume ratio (≤ 0.1)	% Recovery	% Deviation	% Recovery within ± 10%
KRYPTOR CP	2.07	0.24	26.24	0.0728	96.17%	−3.83%	Yes
KRYPTOR CP	14.87	13.57	26.75	0.1	99.21%	−0.79%	Yes
Wondfo	0.98	0.30	25.62	0.0225	117.95%	11.95%	No
Wondfo	4.24	1.30	25.35	0.1	118.32%	18.32%	No
Getein	2.06	0.31	25.87	0.0475	142.37%	42.37%	No
Getein	9.48	6.97	25.92	0.1	123.59%	23.59%	No
Snibe	1.88	0.34	41.87	0.0375	98.13%	−1.87%	Yes
Snibe	13.30	9.52	42.13	0.1	112.56%	12.56%	No

### Method comparison

Table 4 presents the results from correlation calculations and Passing-Bablok regression analyses. When comparing assays from the limit of quantitation (LoQ) to 100 μg/L, the PCT-W, PCT-G, and PCT-S assays showed high Spearman correlations with the PCT-KR assay (respectively, rS = 0.962; rS = 0.976; and rS = 0.975). For the PCT-W assay, Passing-Bablok regression revealed a moderate proportional bias with a positive intercept, indicating some constant bias (equation: PCT-W = 0.724 PCT-KR + 0.056). In contrast, the PCT-G and PCT-S assays had slopes close to 1 and negative constant bias intercepts (equations: PCT-G = 0.94 PCT-KR−0.088 and PCT-S = 0.968 PCT-KR−0.06).

**TABLE 4:** Passing-Bablok regression results and Spearman correlation coefficients for samples from limit of quantitation to 2 μg/L and 100 μg/L, respectively; Cohen’s Kappa values for agreement in the application of LRTI and systemic bacterial infection-sepsis classifications for all samples.

	LoQ to 2 μg/L				LoQ to 100 μg/L				All samples		
		**P-B regression**		**Corr.**		**P-B regression**		**Corr.**	**Classification agreement**		
**Assay**	***N***	**Intercept**	**Slope**	**r_*s*_**	***N***	**Intercept**	**Slope**	**r_*s*_**	***N***	**LRTI K_*C*_**	**Sepsis K_*C*_**
Wondfo	196	0.076	0.663	0.901	273	0.056	0.724	0.962	350	0.78	0.83
Getein	183	−0.060	0.838	0.938	254	−0.088	0.940	0.976	350	0.65	0.87
Snibe	210	−0.069	1.002	0.939	286	−0.060	0.968	0.975	350	0.76	0.92

LoQ, limit of quantitation; P-B, Passing-Bablok; Corr., correlation.

**FIGURE 2:**
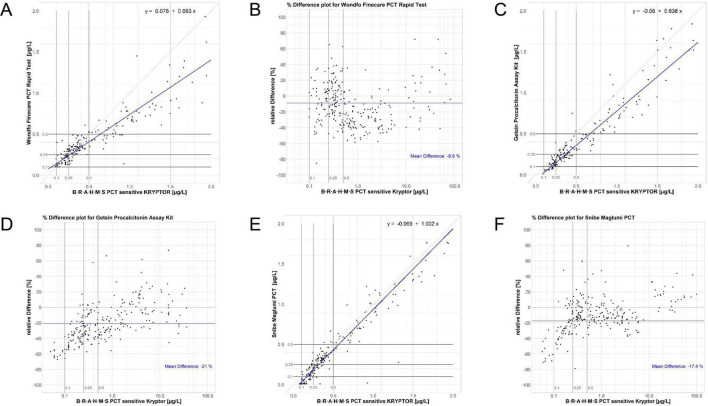
Regression and normalized bias plots for the Wondfo [respectively, **(A,B)**], Getein [respectively, **(C,D)**], and Snibe [respectively, **(E,F)**] assays versus the KRYPTOR. Low-range diagnostic cut-offs are highlighted in all plots, these being 0.1, 0.25, and 0.5 μg/L.

Within the LoQ to 2 μg/L range, assay performances varied. PCT-W and PCT-G exhibited larger proportional bias slopes (equations: PCT-W = 0.663 PCT-KR + 0.076 and PCT-G = 0.838 PCT-KR−0.06, see Figures 2A, C), indicating increased proportional bias at low concentrations. Correlation results for PCT-W, PCT-G, and PCT-S were high (respectively, rS = 0.901; rS = 0.938; and rS = 0.939). The regression for PCT-S (PCT-S = 1.002 PCT-KR−0.069) closely aligned with the results from the LoQ to 100 μg/L range. Overall, analysis of discordances featured in the Passing-Bablok regression plots points to a poorer agreement at low concentrations (see Figures 2A, C, E). Table 4 shows the Kappa coefficient agreement between the assays. The PCT-W assay demonstrated relatively close Kappa values for both LRTI and sepsis classifications (respectively, KC = 0.78 and KC = 0.83). Most misclassifications occurred around the 0.5 μg/L cut-off, with significant increases in bias observed at and below this cut-off, as seen in the Bland-Altman plot (Figures 2A, B). The PCT-G assay had the lowest concordance for LRTI (KC = 0.65) but performed better for sepsis classification (KC = 0.87). Similarly, the PCT-S assay showed better classification agreement for sepsis (KC = 0.92) compared to LRTI (KC = 0.76). Notably, a positive bias trend for higher sample concentrations was observed in the bias plots for both PCT-G and PCT-S (Figures 2D, F).

## Discussion

The PCT-KR assay demonstrated total in-laboratory precision, linearity, and recovery consistent with the manufacturer’s specifications (23). In contrast, all three other assays did not meet the recovery specifications outlined in their instructions for use (21, 22, 24). It should be noted that PCT-W and PCT-G were tested using the PCT-KR standard solution, but the commutability of this standard with PCT-W and PCT-G has not been established. Additionally, PCT-W and PCT-G did not meet the linear correlation values specified in their documentation (21, 22).

While the Kappa coefficients of agreement for sepsis cut-offs were above 0.8 for all three assays, the agreements for lower respiratory tract infection (LRTI) cut-offs were below this threshold, showing only substantial agreement. This was unexpected given that the limits of detection reported by the assay manufacturers were relatively high: 0.1 μg/L for PCT-W and PCT-G, and 0.13 μg/L for PCT-S (21, 22, 24). Negative bias increased for PCT-G and PCT-S at low concentrations (≤ 0.5 μg/L), which contributed to poorer agreement for LRTI cut-offs compared to sepsis cut-offs, due to the presence of additional low-concentration cut-offs in LRTI classifications. The PCT-W assay exhibited the highest proportional and constant bias values in the Passing-Bablok regression for concentrations ranging from the limit of quantification (LoQ) to 2 μg/L, with values of −33.7% and 0.076 μg/L, respectively. Surprisingly, despite these biases, the agreement of PCT-W with the PCT-KR assay was the highest among all assays for LRTI diagnostic cut-offs. Cut-offs used for diagnosing and managing LRTI and sepsis are particularly stringent for the low-range performance of assays. Therefore, a thorough assessment of assay performance must include a close examination of how assays perform with low concentration samples. Regression and Bland-Altman bias plots were instrumental in this evaluation. Both the PCT-G and PCT-S assays exhibited increasingly negative bias values below the 0.5 μg/L cut-off. This bias resulted in the classification of samples into lower concentration categories compared to the predicate assay, leading to potential underestimation of sample concentrations and, consequently, the risk of under diagnosis of infections and inappropriate antibiotic use, especially for LRTI. Moreover, the specificity and sensitivity of PCT-W, PCT-G and PCT-S comparison demonstrated in Supplementary Table 1, indicated that there were specificity and sensitivity variation in those three assays compared to the standard PCT-KR. And the ROC curves to evaluate sensitivity and specificity at critical cut-offs (e.g., 0.15 μg/L, 0.25 μg/L, 0.5 μg/L) is demonstrated at Supplementary Figure 1.

In contrast, the PCT-W assay displayed a wide range of bias values, including both high negative and positive biases below the 0.5 μg/L cut-off. This variability led to the misclassification of samples into both higher and lower concentration ranges around the 0.25 μg/L cut-off, which could adversely affect patient management. Above the 0.5 μg/L cut-off, PCT-W showed an increase in negative bias, leading to further misclassification of samples due to underestimated concentrations. Similar misclassification issues have been reported by Li et al. (26), who compared plasma samples using the Roche Elecsys B⋅R⋅A⋅H⋅M⋅S PCT assay. Discrepancies between our findings and Li et al. (26) may stem from matrix differences (serum vs. plasma) and platform-specific antibody epitope recognition, particularly for PCT-G. When using B⋅R⋅A⋅H⋅M⋅S PCT algorithms, this underestimation could lead to under-diagnosis and unnecessary discontinuation of antibiotics.

Additionally, the total laboratory imprecision for low concentrations (∼0.25 μg/L) was significantly higher for these assays compared to the PCT-KR assay (4.64% for PCT-KR versus 8.38% to 15.67% for the other assays). In conclusion, the analytical performance of the assays at low concentrations compromised their effectiveness for sepsis and LRTI cut-offs, potentially impacting clinical accuracy and safety. Publications discussing the performance of the assays compared in this study are limited, restricting direct comparisons. One study evaluates the PCT-W assay using whole blood with a very small sample size, making comparisons difficult (27). Another publication compares plasma samples tested with Roche Elecsys B⋅R⋅A⋅H⋅M⋅S PCT, PCT-W, and PCT-G, but the reader instruments used in that study differ from those in the present study. This previous work employed Kappa coefficients for single cut-off classifications, reporting high agreement values for the 0.5 μg/L cut-off (PCT-W Kc = 0.8 and PCT-G Kc = 0.88), while PCT-G showed only moderate agreement at the 0.25 μg/L cut-off (PCT-W Kc = 0.8 and PCT-G Kc = 0.49) (26).

A multicenter study testing plasma with the PCT-S assay reported a high proportional bias (+51%) and a negative constant bias (−0.1 μg/L) compared to the PCT-KR. The percent agreement values for patient classifications at specific cut-offs were higher than those in the present study: 0.10 μg/L (96% vs. 92%), 0.25 μg/L (96% vs. 92%), 0.5 μg/L (93% vs. 97%), and 2.0 μg/L (96% vs. 99%). Unfortunately, Kappa coefficients were not reported in this study, and it did not use serum samples, complicating comparisons (28). Another study with a small sample size of 40 serum samples found very low compliance rates for the PCT-S assay at LRTI cut-offs (< 0.10 μg/L: 0%; ≥ 0.1 μg/L to < 0.25 μg/L: 40%; ≥ 0.25 μg/L to ≤ 0.5 μg/L: 14.29%; > 0.5 μg/L: 100%), using the Roche Elecsys B⋅R⋅A⋅H⋅M⋅S PCT as the reference assay (29).

### Limitations

This study is limited by its single-center design, evaluation of only three assays, and absence of longitudinal PCT monitoring for algorithm validation.

## Conclusion

All assays (PCT-W, PCT-G, and PCT-S) demonstrated some level of classification agreement with the PCT-KR reference assay for both sepsis and LRTI cut-offs. However, issues with recovery and poor precision around the 0.5 μg/L cut-off were noted for the PCT-S assay. The PCT-W and PCT-G assays revealed discrepancies with the reference in terms of recovery, imprecision, and linearity. Additionally, all assays showed increased individual sample bias at low concentrations. Underestimation of PCT at 0.25 μg/L (e.g., PCT-G bias: −33%) could lead to delayed antibiotic initiation in 15–20% of LRTI cases, as per Schuetz et al. (30). Conversely, overestimation (e.g., PCT-W bias: +18%) may result in unnecessary antibiotic prescriptions, increasing antimicrobial resistance risks. Therefore, the analytical performance of PCT-W, PCT-G, and PCT-S around the low-end cut-offs for LRTI and sepsis was found to be inadequate for accurate diagnosis and management.

In summary, despite showing classification agreement with the reference assay, the suitability of these assays for use with B⋅R⋅A⋅H⋅M⋅S PCT algorithms is compromised by various analytical performance issues. This raises concerns about the accuracy and safety of the evaluated assays. Further investigation is needed to assess their performance in clinical settings and to explore their diagnostic accuracy and clinical impact.

## Data Availability

The raw data supporting the conclusions of this article will be made available by the authors, without undue reservation.
