# Supplementary material for: Analytical evaluation of the performances of point-of-care and benchtop procalcitonin assays in comparison with the B⋅R⋅A⋅H⋅M⋅S PCT sensitive KRYPTOR assay
Source: Front Med (Lausanne). 2025 Jun 17;12:1487557. doi: 10.3389/fmed.2025.1487557 (PMC12209313; doi:10.3389/fmed.2025.1487557)
Supplement: Supplementary file 1 [file Table_1.docx]

*Table S1. LRTI four cell table, summarized sensitivity and specificity comparison of PCT-W, PCT-G and PCT-S to the standard KRYPTOR method in cutoff value 0.25 µg/L*

| PCT-W vs. KRYPTOR | **KRYPTOR≥0.25 µg/L** | **KRYPTOR <0.25 µg/L** |
| --- | --- | --- |
| **PCT-W ≥0.25 µg/L** | 146 | 1 |
| **PCT-W <0.25 µg/L** | 0 | 203 |
| **Sensitivity:** | 100% | |
| **Specificity:** | 99.5% | |
| PCT-G vs. KRYPTOR | **KRYPTOR≥0.25 µg/L** | **KRYPTOR <0.25 µg/L** |
| **PCT-G ≥0.25 µg/L** | 200 | 5 |
| **PCT-G <0.25 µg/L** | 1 | 144 |
| **Sensitivity:** | 99.5% | |
| **Specificity:** | 96.6% | |
| PCT-S vs. KRYPTOR | **KRYPTOR≥0.25 µg/L** | **KRYPTOR <0.25 µg/L** |
| **PCT-S ≥0.25 µg/L** | 200 | 5 |
| **PCT-S <0.25 µg/L** | 1 | 144 |
| **Sensitivity:** | 99.5% | |
| **Specificity:** | 96.6% | |
